# Technical skills in complex tennis situations: Dutch talented players U15 compared to players U17

**DOI:** 10.3389/fspor.2023.1107740

**Published:** 2023-03-01

**Authors:** Nikki S. Kolman, Barbara C. H. Huijgen, Chris Visscher, Marije T. Elferink-Gemser

**Affiliations:** ^1^ Center for Human Movement Sciences, University Medical Center Groningen, University of Groningen, Groningen, Netherlands; ^2^ Knowledge Center for Sport & Physical Activity, Utrecht, Netherlands; ^3^ Department of Psychology, University of Groningen, Groningen, Netherlands

**Keywords:** technique, racket sports, expertise, cognition, precision, performance

## Abstract

**Introduction:**

Technical skills in complex situations appear crucial for progress towards elite tennis performance. However, it is unknown how these skills develop in different age categories in a group of talented youth players. The aim of this study is to evaluate possible differences in technical skills among Dutch talented youth tennis players U15 compared to U17.

**Methods:**

A total of 19 players (12 males, 7 females; age 14.6 ± 1.4 years) were tested on ball speed, accuracy, percentage errors and spin rate using the on-court Dutch Technical-Tactical Tennis Test. With a ball machine, four games were simulated which were either fixed (game 1 and game 2) or variable (game 3 and game 4), depending on the complexity of the task. Each game consisted of two offensive, two neutral and two defensive rallies, representing different tactical situations.

**Results:**

A two-way ANOVA revealed a statistically significant interaction between the effects of age category and sex for ball speed (*F*(1,15) = 5.472, *p* = 0.034, *η*^2^ = 0.267), indicating that males U17 produced higher ball speed compared to males U15, whereas no differences were found between females U15 and U17. A one-way ANCOVA showed that, regardless of sex, players U17 scored significantly higher on accuracy than players U15 (*F*(1,16) = 5.021, *p* = 0.040, *η*^2^ = 0.239). No differences were found between players U15 and U17 for spin rate and percentage errors (*p* > .05), although there was a medium to large effect size for males U17 to produce higher spin rates compared to males U15. A closer examination of accuracy revealed that players U17 scored significantly higher compared to players U15 in game 4 (*F*(1,17) = 6.358, *p* = .022, *η*^2^ = .272) and in defensive situations (*F*(1,17) = 9.602, *p* = .007, *η*^2^ = .361).

**Discussion:**

In conclusion, the results of the current study suggest that technical skills, especially ball speed for males and accuracy in complex situations for both males and females, continue to develop in adolescence in talented tennis players. There is an increased understanding about underlying technical skills that contribute to progress towards elite tennis performance. To effectively develop technical skills, coaches are encouraged to design specific practices where these skills are performed in complex situations under high cognitive and temporal pressure.

## Introduction

Many structured talent development programs have been developed for sports, including tennis (1, 2). National tennis associations provide specialized training programs with the aim of developing and perfecting tennis performance. Offering the best facilities, training and guidance is thus a priority for associations in order to develop talented players optimally. Unfortunately, our understanding of talent development processes is rather limited and it is difficult to provide specific recommendations for tennis associations (3). A thorough understanding of tennis-specific skills during a player's adolescence is required to facilitate the development of talents performing at a level where details make the difference.

Outstanding technical skills are considered essential for performance in sports. Most of the studies in a recent systematic review found that technical skills discriminate between performance levels, explain past performance or predict future performance (4). Studies on tennis-specific technical skills underline that players at a higher performance level outscore players at a lower performance level on measures such as ball speed, percentage errors and accuracy (5). An increased ball speed reduces the time for an opponent to return the ball successfully (6, 7). The amount of errors seems particularly important for reaching professional level, as the error rate is lower among professional players compared to elite youth players (8). To be in control in a match, players should also hit their strokes with sufficient accuracy as hitting the ball to a specific location on the court allows them to keep the ball far enough from their opponents to produce a winner or cause the opponent to make an error (9). Spin rate, however, may be equally important, because the amount of spin imparted to the ball affects its ball trajectory. This is useful to overcome constraints of the game (i.e., net and court boundaries) or for a tactical advantage (10).

The relevance of technical skills for youth tennis performance was confirmed by a recent prospective study showing that ball speed and accuracy measured under 14 years (U14) were significant predictors of tennis performance at the same time and 4 years later (11). Technical skills were assessed with the Dutch Technical-Tactical Tennis Test (D4T), a reliable and valid on-court test (12). Games were simulated which were either fixed or variable. In the fixed situations, players needed to direct their strokes to predetermined target areas, whereas in the variable situations the players were required to consider the direction of their strokes (e.g., respond to an imaginary opponent). Variable situations were considered more complex compared to fixed situations, due to the presumed higher cognitive load. More in depth-analyses of this prospective study revealed that the ability to maintain accuracy in variable situations, not in fixed situations, was considered essential to reach the elite level under 18 years (U18). In other words, players who reached the elite level U18 were more accurate in variable situations in their younger years (i.e., U14) compared to lower performing players U18. However, how these technical skills develop during adolescence, especially from the age of 12–16 years, and what important technical changes take place during this period remains open to debate. Adolescence is regarded as a key developmental phase in the course of talented players’ careers. Development occurs in combination with physical change, including puberty, the pubertal growth spurt, and accompanying maturational changes (13). By exploring the technical skills of talented players in different age categories, we may acquire a better understanding of underlying technical skills that contribute to progress towards elite tennis performance. Knowledge about the important technical changes during adolescence may be of value for the adaptation of talent development programs.

From a constraints-led perspective, technical performance emerges from the interaction between the person (e.g., anthropometry, physical skills), the environment (e.g., court surface, type of competition) and the task at hand (e.g., complexity, intensity) (14, 15). Through systematically manipulating constraints it is possible to construct and mimic a tennis-specific situation. With the D4T, task constraints are manipulated by changes in the complexity of the task. From the literature it is apparent that if the complexity of the task increases, there is a decrease in technical performance in a range of sports including ice hockey, rugby and soccer (16–18). By means of simulating fixed and variable situations, the D4T allows tennis players to experience technical demands in situations of different complexity. Another way to adjust the complexity in the D4T is by changes in time constraints. The impact of time constraints on tennis performance is reflected by simulating offensive, neutral and defensive situations in the D4T. Players need to make quick and accurate decisions in order to perform accurately under high time pressure (19). In a defensive situation, there is less time for anticipating the direction of an opponents’ stroke and keeping the accuracy of strokes high compared to an offensive situation where players are in control of the rally (20). The speed-accuracy tradeoff is highlighted in a group of youth tennis players with less accuracy in defensive compared to offensive situations (12). Given that technical skills are always executed in a particular context, we must consider the tennis-specific context when examining the technical skills in a group of talented youth players.

Technical skills in complex situations appear crucial for progress towards elite tennis performance, however, it is unknown how these skills develop in different age categories in a group of talented youth players. Therefore, our aim of this study is to evaluate possible differences in technical skills of Dutch talented youth tennis players under 15 (U15) compared to under 17 years (U17). We hypothesized that (a) players U17 have superior technical skills compared to players U15 and (b) differences between players U17 and U15 are most pronounced in complex situations (i.e., variable and defensive situations).

## Method

### Ethical approval

Ethical approval for this research protocol (PSY-1819-S-0262) was obtained from the Psychology Department of the University of Groningen (Groningen, Netherlands, September 19th, 2019). We obtained advanced written informed consent or assent from all players and advanced written informed consent from parents or legal guardians of all players under 16 years of age (the legal age for giving consent in the Netherlands).

### Participants

Nineteen youth players between 12 and 17 years old (12 males, 7 females; age 14.6 ± 1.4 years) participated in this study. All participants were within the national high-performance program of the Royal Dutch Lawn Tennis Association (KNLTB). According to their year of birth, males were ranked between position 2 and 14 on the national ranking list of the KNLTB, while females were ranked between position 1 and 5. Table 1 shows the age, anthropometric characteristics, tennis history, tennis practice and additional physical practice for players U15 and U17 and males and females separately.

**Table 1:** Descriptive statistics (mean ± SD) of talented youth tennis players (*n *= 19).

	U15			U17		
	Male (*n *= 7)	Female (*n *= 4)	Total (*n *= 11)	Total (*n *= 8)	Male (*n *= 5)	Female (*n *= 3)
Age (years)	13.7 ± 0.7	13.2 ± 0.5	13.5 ± 0.6	16.0 ± 0.5	16.1 ± 0.6	15.7 ± 0.2
Height (cm)	168.2 ± 12.9	166.5 ± 5.8	167.6 ± 10.5	176.3 ± 5.8	177.7 ± 3.5	174.1 ± 9.1
Weight (kg)	52.1 ± 11.8	51.1 ± 5.1	51.7 ± 9.6	67.4 ± 6.2	69.2 ± 5.4	64.3 ± 7.4
Maturity offset (years)	−0.2 ± 1.4	1.4 ± 0.6	0.4 ± 1.4	2.3 ± 0.7	2.0 ± 0.4	2.7 ± 1.0
Age starting tennis (years)	6.4 ± 1.8	5.5 ± 0.6	6.0 ± 1.5	4.3 ± 1.5	4.3 ± 1.7	5.7 ± 1.2
Tennis experience (years)	7.4 ± 1.5	7.7 ± 1.0	7.5 ± 1.3	11.8 ± 1.6	12.1 ± 1.9	11.3 ± 1.4
Tennis practice (hours/week)	11.8 ± 2.5	10.9 ± 1.5	11.5 ± 2.2	14.5 ± 2.4	14.5 ± 2.4	14.5 ± 3.0
Physical practice (hours/week)	3.8 ± 1.1	4.5 ± 0.5	4.0 ± 1.0	5.3 ± 1.0	5.1 ± 0.9	5.7 ± 1.2

### Measures

#### Anthropometry

Anthropometric data were obtained, which included body height, sitting height and body mass. Players’ body height and sitting height were measured with a SECA height tape instrument to the nearest 0.1 cm (SECA, model 206, Seca Instruments, Ltd., Hamburg, Germany). Players were standing with bare feet against the wall (or were sitting on a bench for sitting height) and were asked to take a deep breath and to hold it. Body mass was measured to the nearest 0.1 kg (UWE, model ATM B150, Universal Weight Enterprise Co., Ltd., Taiwan). Leg length was calculated by subtracting sitting height from body height. Maturity status was estimated by the non-invasive method of calculating the age at peak height velocity using sex-specific predictive equations (21).

#### Technical skills

**Figure 1:**
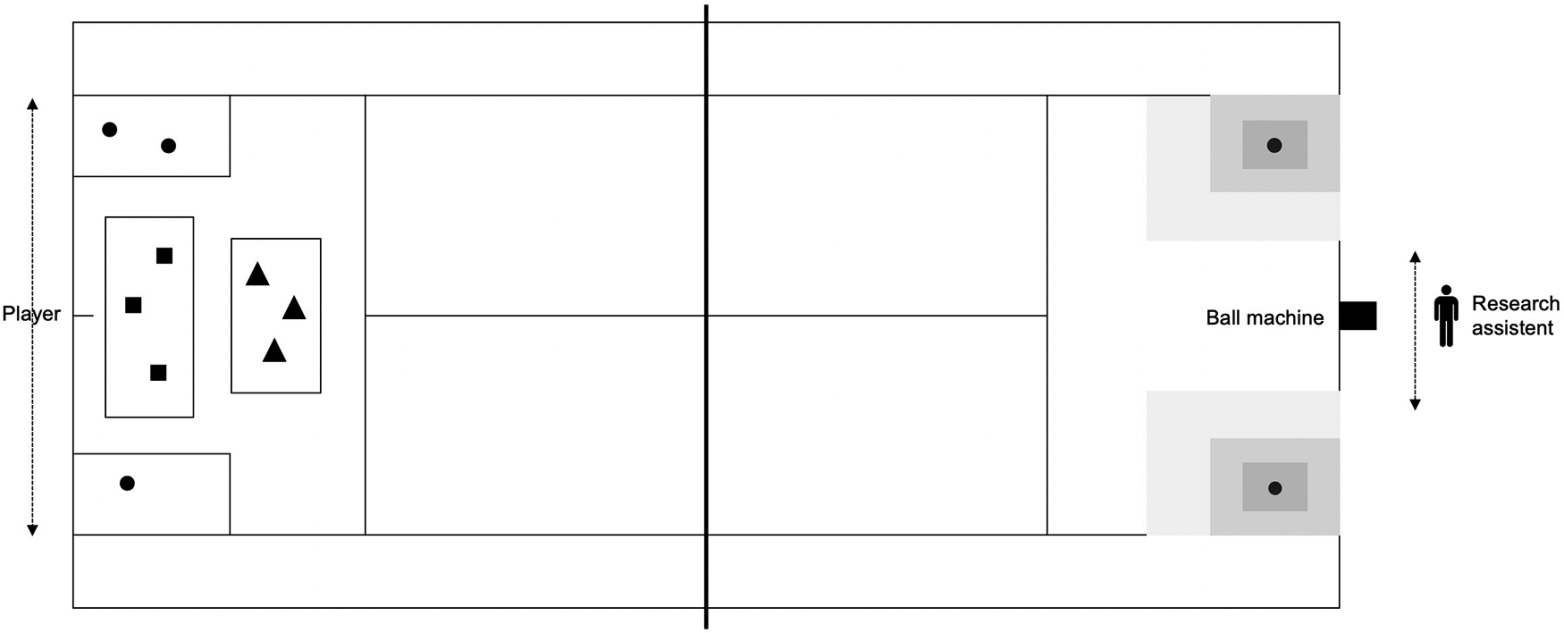
Illustration of the D4T with various tactical situations. This figure shows the test situation of the D4T with various tactical situations. The symbols represent the three ball projections in an offensive (▴), neutral (▪) and defensive (●) tactical situation.

Ball speed, accuracy, percentage errors and spin rate were measured with the Dutch Technical-Tactical Tennis Test (D4T), a reliable and valid instrument to measure technical skills in youth players (12). The D4T requires players to hit 72 balls, grouped in four games of six rallies, in which each rally includes three strokes fed by a ball machine. Each game consists of two offensive, two neutral and two defensive rallies, representing different tactical situations as displayed in Figure 1. The difficulty of the ball projections was slightly increased compared to the original D4T, making it more suitable for a group of talented youth players. Offensive rallies consist of three ball projections just beyond the service line. Neutral rallies comprise of three ball projections to the area around the middle of the court a half to one meter before the baseline, and defensive rallies includes three ball projections to the sideline and beyond the service line. The different tactical situations occurred in random order in each game.

The various games have increasing complexity. In the first and second game, players have to return their strokes to the left target area (deuce side) and right target area (advantage side), respectively (Figure 1). In the third game, players have to alternate their strokes between the left and right target area. For example, if players direct their strokes to the left-right-left target area in the first rally, they should aim their strokes to the right-left-right target area in the second rally. In the fourth game, players have to return their strokes to the left or right target area, as indicated by a simulated opponent (research assistant) who moves either 1.5 meters to the left or right side of the court. Hence, players have to return their strokes to the opposite side of the side where the opponent is moving to. This is a modification from the original D4T where the target area in the fourth game was determined by lights which turned red either on the left or right side of the court. The simulated opponent was used instead of lights to increase the ecological validity of the D4T. The conditions in the first and second game were more fixed compared to the variable and complex conditions in the third and fourth game. During the test, players were allowed to rest for 15 s in between the rallies and 90 s after three games, which was similar to match play. More detailed information on the D4T has been reported previously (12).

Technical skills were recorded with PlaySight SmartCourt, a system for video-review and analytics and equipped with 10 on-court cameras. This system allows for the valid registration of ball speed, ball placement, spin rate and the registration of session video material (Playsight, 2015). For accuracy, a total of nine, six and three points were awarded to balls landing inside the small, middle and large target area, respectively (Figure 1). One point was awarded to balls landing outside the target areas, but still in the court on the correct side (determined by the given game situation). Balls landing in the wrong side of the court, outside the singles lines or in the net, were awarded with zero points. Percentage errors was calculated as the number of faults divided by the total number of strokes multiplied by hundred.

### Procedures

All measurements took place at the National Training Center of the KNLTB in Amstelveen in the Netherlands. Measurements took place on a hard-court indoor tennis court with PlaySight SmartCourt system for video-review and analytics using 10 on-court cameras. Before the D4T, players performed a warm-up of 10 min, including 5 min of hitting groundstrokes. Players were alternately tested with the remaining players conducting a training session at low to medium intensity. Measurements took place in the morning or afternoon (10.00 a.m. to 18.00 p.m.), depending on players’ time of training. Participants were fed with moderately used tennis balls (Dunlop Fort Max TP) by a manually programmed ball machine (Promatch SmartShot Xtra, Mubo, Gorinchem). Participants used their own tennis racket during the test protocol. Before the measurements, a research assistant was trained to move 1.5 meters to either the left or right side of the court just after the ball was fed by the ball machine. The research assistant moved according to a predetermined program, with half of the movement being to the left and right, respectively.

### Data analysis

For the statistical analyses, we used SPSS Statistics for Mac, version 28 (IBM Corp., Armonk, N.Y.). For all significance tests, we used an *α*-level of 0.05. We screened the data to ensure variables met the assumptions necessary for the use of parametric statistics before data analysis. We performed a one-way ANCOVA with age category as grouping factor (U15 versus U17) for each technical skill separately (i.e., ball speed, accuracy, percentage errors and spin rate), whilst controlling for sex which we considered a covariate. When heterogeneity of regression slopes was found, we performed a two-way ANOVA to analyze the effect of age category and sex on the relevant technical skill. We considered an effect size of *η*^2^ = 0.01 as small, *η*^2^ = 0.06 as medium and *η*^2^ = 0.14 as large (22). In the case of a significant covariate and for the technical skills that were statistically different between players U15 and U17, we performed additional analyses. We conducted one-way ANOVAs to further unravel differences between age categories for the relevant technical skills in complex situations. First, we assessed differences between players U15 and U17 for the relevant technical skill in fixed and variable game situations. Second, we measured differences between players U15 and U17 for the relevant technical skill in different tactical situations.

## Results

Table 2 illustrates the mean scores of technical skills for players U15 and U17 and males and females separately. A one-way ANCOVA revealed a significant interaction between age category and the covariate sex for ball speed, indicating that the assumption of homogeneity of regression slopes was violated. Therefore, a two-way ANOVA was performed to analyze the effect of age category and sex on ball speed. There was a statistically significant interaction between the effects of age category and sex [*F*(1,15) = 5.472, *p* = 0.034, *η*^2^ = 0.267]. Simple main effects analyses showed no statistically significant effect of age category on ball speed [*F*(1,15) = 2.128, *p* = 0.165, *η*^2^ = 0.124], while there was a statistically significant effect of sex on ball speed [*F*(1,15) = 8.568, *p* = 0.010, *η*^2^ = 0.364]. Males U17 produced higher ball speed compared to males U15 [*F*(1,10) = 11.017, *p* = 0.008, *η*^2^ = 0.524], while no differences were found between females U15 and U17 [*F*(1,5) = 0.250, *p* = 0.638, *η*^2^ = 0.048].

**Table 2:** Descriptive statistics of technical skills (mean ± SD) and differences between talented tennis players U15 and U17.

	U15			U17		
	Male (*n *= 7)	Female (*n *= 4)	Total (*n *= 11)	Total (*n *= 8)	Male (*n *= 5)	Female (*n *= 3)
Ball speed (kmh)	95.7 ± 7.2*	93.8 ± 5.7	95.0 ± 6.5	101.3 ± 10.0	107.4 ± 3.6*	91.1 ± 8.8
Accuracy (pts)	2.5 ± 0.5	2.5 ± 0.5	2.5 ± 0.5*	2.9 ± 0.3*	2.9 ± 0.3	2.9 ± 0.3
Errors (%)	27.8 ± 10.0	26.4 ± 6.5	27.3 ± 8.5	26.8 ± 5.5	26.5 ± 4.1	27.3 ± 8.4
Spin rate (rpm)	840.7 ± 243.9	659.6 ± 105.9	774.8 ± 217.7	884.6 ± 287.7	1015.9 ± 273.9	665.9 ± 157.3

* *p* < 0.05 significantly different between players U15 and U17.

A one-way ANCOVA revealed a significant main effect of age category on accuracy after controlling for sex [*F*(1,16) = 5.021, *p* = 0.040, *η*^2^ = 0.239]. No differences were found between players U15 and U17 for spin rate [*F*(1,16) = 1.221, *p* = 0.286, *η*^2^ = 0.071] and percentage errors [*F*(1,16) = 1.2711, *p* = 0.885, *η*^2^ = 0.001], although sex was found a significant covariate for spin rate [*F*(1,16) = 5.861, *p* = 0.028, *η*^2^ = 0.268]. No differences were found between females U15 and U17 [*F*(1,5) = 0.004, *p* = 0.952, *η*^2^ = 0.001] and males U15 and U17 [*F*(1,10) = 1.363, *p* = 0.270, *η*^2^ = 0.120] for spin rate, although the medium to large effect size for males indicates that males U17 produced higher spin rates than males U15.

### Accuracy in tennis-specific situations

**Figure 2:**
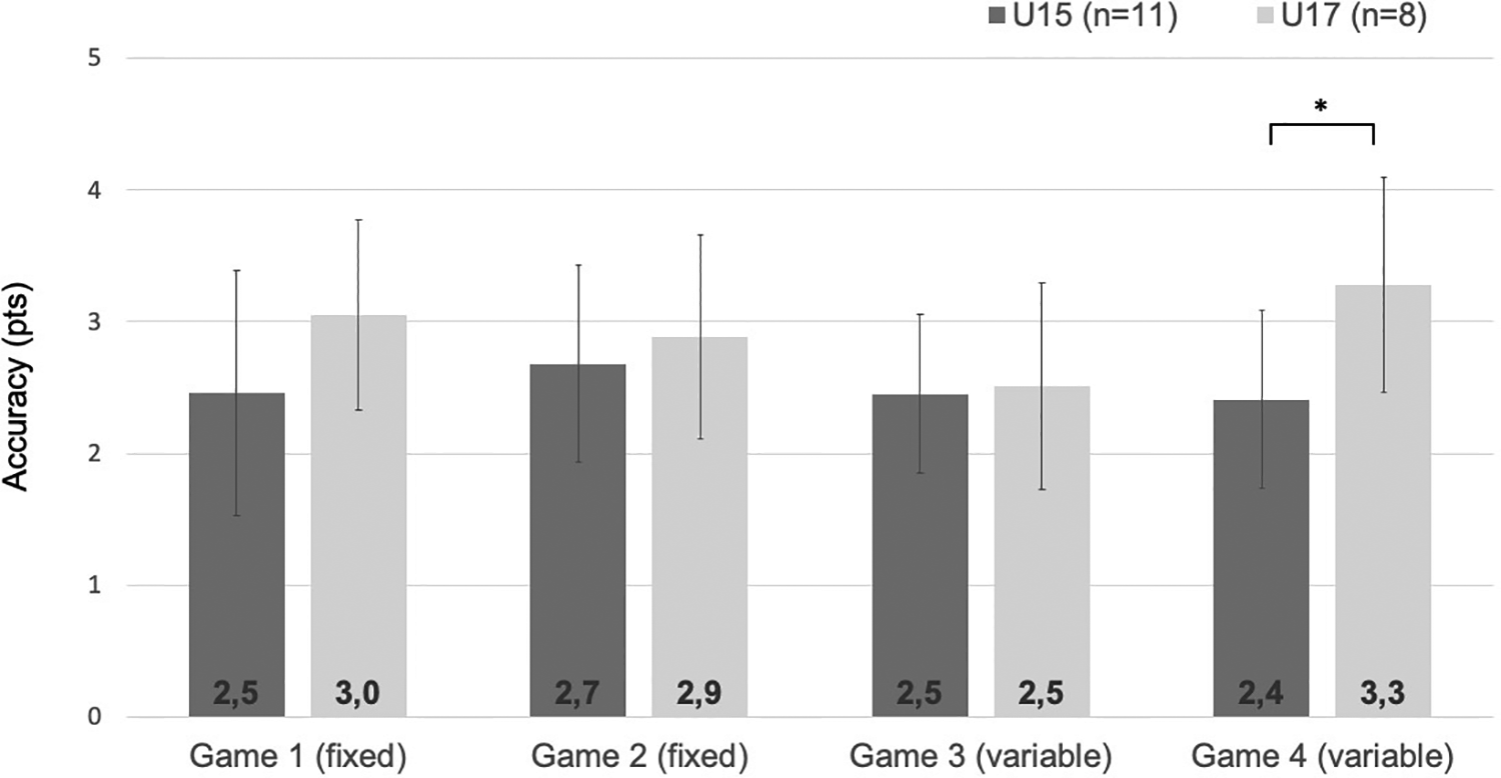
Accuracy in fixed and variable games for players U15 and U17. This figure shows the mean accuracy in various game situations (errors bars represent standard deviations of the mean); *p < 0.05 significant difference between players U15 and U17 for accuracy.

**Figure 3:**
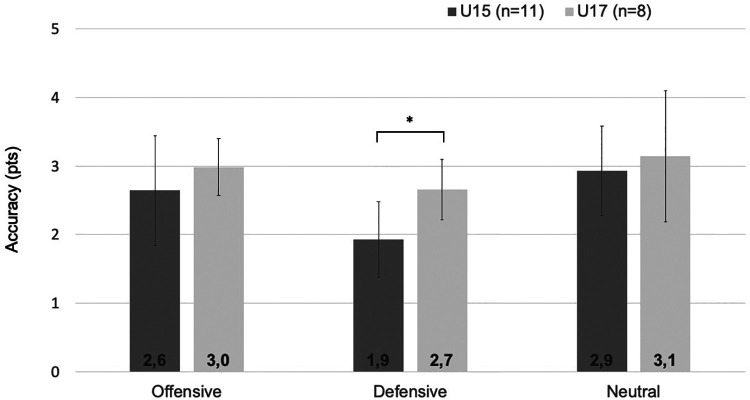
Accuracy in tactical situations for players U15 and U17. This figure shows the mean accuracy in tactical situations (errors bars represent standard deviations of the mean); *p < 0.05 significant difference between players U15 and U17 for accuracy.

Based on the significant difference between age categories for accuracy, we performed additional analyses for accuracy in complex situations. Figures 2, 3 show the accuracy for players U15 and U17 in fixed and variable game situations and different tactical situations, respectively. A significant difference was found between players U15 and U17 on accuracy in game 4 [*F*(1,17) = 6.358, *p* = 0.022, *η*^2^ = 0.272] and accuracy in defensive situations [*F*(1,17) = 9.602, *p* = 0.007, *η*^2^ = 0.361].

## Discussion

To evaluate possible differences in technical skills among talented tennis players in different age categories, players of the Dutch national high-performance program U15 and U17 were compared on different technical skills. Males U17 produced higher ball speed compared to males U15, while no differences were found between females U15 and U17. A difference was found between age categories for accuracy for both male and female players, with players U17 being more accurate than players U15. A closer examination of accuracy demonstrates that players U17 scored higher in complex situations than players U15, given the higher accuracy in the variable game 4 and in defensive situations. These findings were in line with our hypotheses and suggest that technical skills, especially ball speed for males and accuracy in complex situations for both males and females, continue to develop in adolescence in a group of youth talented tennis players.

According to the constraints-led approach, changing task constraints requires an adaptation of the current motor behavior. By differences in task complexity, players were forced to deal with various situations in order to maintain or improve the accuracy of their strokes. In line with earlier research, our findings reveal that under increased task complexity (i.e., high temporal and cognitive pressure), the older and more experienced players were better able to maintain their accuracy than their younger and less experienced counterparts (23). Tennis players are confronted with situations in which motor and cognitive tasks have to be executed simultaneously (24, 25). For example, players need to anticipate the next ball, recall strategies and play the ball with adequate speed and accuracy while being aware of their opponents’ strengths and weaknesses. Usually, performance decreases under increased task complexity. Unlike the fixed situations of the D4T, the variable situations required players to consider the direction of their next ball, possibly increasing the demands on attention and working memory (26, 27). This is also apparent from the results of a previous study with the D4T in which future elite players (mean age 13.7 ± 0.5 years) were able to maintain their accuracy throughout the game situations, while competitive players (mean age 13.3 ± 0.5 years) became less accurate during the variable, more complex situations (11). Both players U15 and U17 were able to maintain their accuracy throughout the game situations, possibly due to their higher performance level compared to the competitive players in the previous study. Where players U17 were more accurate in game 4 than players U15, no differences between these age categories were found in game 3. An explanation for these findings might be related to the less pronounced task complexity in game 3 compared to game 4 where players needed to look at the other side of the net to see which side the simulated opponent moved in order to play the ball to the opposite side. The accuracy of players U17 even seemed to benefit from the increased task complexity in game 4 as indicated by the slightly higher accuracy compared to the others game situations. Due to more years of tennis experience, players U17 might have developed a higher degree of automatization, resulting in a greater resistance to skill decrement under more complex situations than players U15 (23, 28). While it is uncommon for players, especially novices, to perform more accurately in variable than in fixed situations, previous research has shown increased performance in complex situations in experienced hockey players (29). One possibility is that the diversion of attention to another task (e.g., focusing on the simulated opponent) attenuates disruptive conscious processing of movements that can occur in fixed situations.

In contrast to players U17, players U15 were unable to maintain their accuracy under high temporal demands, imposed by ball projections to the sidelines of the court in the defensive situations. The decrease in accuracy in players U15 suggests that the task complexity in the defensive situation might have been too high, causing them to play less accurately due to the greater information processing load (30). In neutral and offensive situations, the task complexity is relatively low, remaining substantial attentional capacity for additional tasks (e.g., focusing on the next ball projection). However, as the temporal pressure increases, greater attention is required to be devoted to maintain stroke accuracy, resulting in reduced processing capacity for anticipating the next ball in the defensive situation. Another explanation for players U17 to be more accurate in defensive situations than players U15 might be related to differences in anthropometry and physical skills such as sprint speed (31, 32) and agility (33). During adolescence, there is an increase in height and players develop more strength and power (13). In the present study, players U17 were taller, heavier and more mature than players U15. Individual differences in growth and maturation, and associated increases in running speed and agility, could translate into an advantage for older youth players in defensive situations.

There was an interaction effect between age category and sex for ball speed, indicating that males U17 produced higher ball speed compared to males U15, while no differences were found between females U15 and U17. These findings were not surprising, given that the maturational time course of males and females is quite different (13). On average, females mature earlier than males. Several studies have shown a relationship between ball speed in groundstrokes and anthropometric factors such as height, weight and maturity status (6, 7, 11). In the present study, females U15 have already experienced their growth spurt as opposed to males U15. During the pubertal transition from early through mid-adolescence, males become taller, heavier and stronger, increasing the differences between males U17 and males U15 on outcomes related to anthropometry and physical skills, such as ball speed. This may also apply to spin rate, given the significant main effect of sex and the medium effect size of age category. Males generated more spin than females, and the medium to large effect size for males indicates that males U17 produced higher spin rates compared to males U15. The effect of anthropometry and physical skills on spin rate merits further investigation, but earlier research studying the mechanics of spin rate also mention the impact angle and racket speed as factors affecting spin rate (34).

There are a few strengths and weaknesses to consider. The design of the D4T provides interesting insights for tennis performance, however it is not completely representative of tennis performance demands. Players were forced to direct their strokes to a specific side of the court, depending on the fixed or variable game situation. The location of the ball projections has impacted the direction of players’ stroke, which was either more cross-court or down the line. Changing the ball angle of a ball projected to the side line, by attempting to play it down the line, possibly increases the amount of lateral errors (35). In actual tennis competition, players are free to decide the direction of their strokes, which may result in a different amount of errors than during the D4T. Another weakness related to the lack of representativeness is the use of a ball machine, where players cannot use relevant kinematic information from the opponent (e.g., distal cues from arm and racket) to anticipate the direction of strokes (36, 37). Returning strokes from a ball machine could result in different swing timing and movement coordination, limiting the generalization of the results (38). However, the use of a ball machine allows for the reliable and valid comparison of technical skills between age categories due to the standardized test design. Another strength of this study was the use of a homogeneous group of talented players, with all participants playing at the highest level in their age category in the Netherlands. Understanding the underlying technical skills of this sample can help optimize talent development programs. Future studies should examine how technical skills measured with the D4T, particularly accuracy in complex situations, relate to on-court tennis performance under high temporal and cognitive pressure. The association of on-court test performance with match activities is considered a feasible approach for evaluating ecological validity (39).

The present cross-sectional study provides insight into the technical differences between players U15 and U17, increasing the understanding of underlying technical skills that contribute to progress towards elite tennis performance. However, the actual process of technical development is unknown and it is unclear whether players U15 improve their skills, and specifically accuracy in complex situations, to the current level of players U17 in 2 years. Differences between these age categories may still exist due to the earlier age of starting tennis, more years of tennis experience and higher amount of training hours for players U17. In future studies, a longitudinal study design is advised to determine the actual process of technical development over time in a group of talented tennis players.

In conclusion, the results of the current study suggest that technical skills, especially ball speed for males and accuracy in complex situations for both males and females, continue to develop in adolescence from U15 to U17 in a group of youth talented tennis players. This study increases the understanding of underlying technical skills that contribute to progress towards elite tennis performance. To effectively develop technical skills, coaches are encouraged to design specific practices where these skills are performed in situations under high cognitive and temporal pressure.

## Data Availability

The raw data supporting the conclusions of this article will be made available by the authors, without undue reservation.
